# Effect of fibrin glue on corneal lamellar healing and how it correlates to biomechanical properties: biomechanical wavefront analysis and confocal study

**DOI:** 10.1186/s40662-016-0046-6

**Published:** 2016-06-01

**Authors:** Almamoun Abdelkader

**Affiliations:** Department of Ophthalmology, Faculty of Medicine, Al-Azhar University, Cairo, Egypt; Ophthalmology Department, Abha Private Hospital, P.O Box 1794, Abha, Kingdom of Saudi Arabia

**Keywords:** Wound healing, Corneal hysteresis, Corneal resistance factor, Ocular Response Analyzer (ORA), Confocal microscopy

## Abstract

**Background:**

To evaluate, using a rabbit model, the influence of the wound healing process at the flap edge on corneal biomechanics after sutured, glued, and non-augmented microkeratome flaps.

**Methods:**

Unilateral 160 μm thick laser in situ keratomileusis (LASIK) flaps using a mechanical microkeratome were performed on the corneas of the left eyes of 36 rabbits. Animals were then divided into 3 groups of 12 rabbits each: A: the flaps were glued with human fibrin tissue adhesive (Tisseel); B: the flaps were sutured; and C: the flaps were allowed to heal without the use of sutures or glue (non-augmented). The contralateral eyes served as controls. Reichert ocular response analyzer (ORA) was used to measure corneal hysteresis (CH), corneal resistance factor (CRF), Goldmann-correlated intraocular pressure (IOPg) and cornea-compensated IOP (IOPcc) at 6 weeks and 3 months postoperatively. In vivo confocal microscopy (IVCM) was also used to study the corneal wound healing process in all groups.

**Results:**

Both mean CH and mean CRF were significantly higher in sutured and glued groups compared with the non-augmented group at 6 weeks and 3 months postoperatively (*P* **<** 0.0001). No statistically significant difference in corneal biomechanics was found between controls and groups A and B at any time points. Activated keratocytes were detected at the wound edge and peripheral flap interface in sutured and glued groups.

**Conclusion:**

The healing process at the wound edge is critical for optimal corneal integrity. Fibrin glue may serve as a safe and effective substitute to sutures in enhancing the corneal flap edge healing response and in increasing its mechanical strength.

## Background

In post-laser in situ keratomileusis (LASIK)  ectasia, the lamellar cut and excimer laser ablation lead to a state of biomechanical failure with an inability to support the continuous stresses caused by intraocular pressure (IOP), extraocular muscle action, blinking, eye rubbing, and other forces [1]. Multiple risk factors have been implicated in the development of corneal ectasia. Among them are abnormal or suspicious topography suggestive of pre-existing forme fruste keratoconus and an excessive resection of corneal tissue that makes the cornea mechanically unstable. However, in some cases none of these risk factors are identified. A wound-healing process characterized by absence of significant fibrosis and myofibroblasts at the wound edge in the flap interface was noted in all keratectatic eyes. In most reports, eyes develop a progressive central or inferior corneal steepening associated with a significant increase in myopia and a progressive thinning of the cornea [2].

The introduction of the Intralase femtosecond laser to create the LASIK flap has been an important milestone in dispelling the myth of the never-healing flap. Laboratory tests show that flap adhesion is significantly stronger when created with a femtosecond laser. Femtosecond assisted laser decreases the incidence of iatrogenic keratectasia, when compared with microkeratome-assisted procedures, however, few cases of ectasia as well as visually significant epithelial ingrowth have been reported after femtosecond assisted LASIK [3]. Traditional mechanical microkeratome for LASIK flap creation has been shown to produce unpredictable cut depths and variable flap thickness [4, 5]. The corneal flap created with a mechanical microkeratome is a meniscus-shaped flap, meaning it is thinner in the center with a beveled flap edge that produces almost no healing response. Alternatively, the femtosecond laser produces a planar flap with uniform thickness from edge to edge thereby inducing a stronger stromal healing response [6].

Flap adherence problems (dislocations) after LASIK can occur with eye rubbing, tight eyelids, or under dry conditions. Fibrin adhesive glue and sutures have been used to reattach the flap and to prevent epithelial ingrowth. Fibrin adhesive has been placed in the flap interface with good success. Sutures have been used to secure the flap in cases of repeated dislocations or significant and persistent flap striae, however, suturing is a time consuming task in ophthalmology and suture induced irritation, irregular astigmatism and redness are frequent problems. To prevent these complications, ophthalmic surgeons are switching to sutureless surgery [7].

This study was designed to evaluate, in a rabbit model, the influence of the wound healing process at the flap edge and flap interface on corneal biomechanics after sutured, glued, and non-augmented microkeratome flaps using the Reichert ocular response analyzer (ORA) and a confocal microscope. This study is the first to examine this issue as a step forward approach to preventing ectasia through inducing stronger corneal healing after a lamellar cut. This research will have important implications for both refractive and lamellar surgery.

## Methods

Thirty-six New Zealand white rabbits weighing 3.0 to 4.0 kg were used in this study. The animals were kept under appropriate dietary conditions. The animals were housed in wire-bottomed individual cages at room temperature (air-conditioned, 22–26 °C) on a 12-h light–dark cycle. All animal studies were approved by the LSU Health Sciences Center Institutional Animal Care and Use Committee. All experiments adhered to the Association for Research in Vision and Ophthalmology (ARVO) resolution on the use of animals in research.

### Anesthesia procedure

Food was withheld from the animals for 12 h before the operation. General anesthesia was performed intramuscularly with a combination of 6 mg/kg xylazine hydrochloride (Rompun; Bayer AG, Leverkusen, Germany) and ketamine hydrochloride (Ketalar, 50 mg/mL, Pfizer; 50 mg/kg body weight). Before the operation, a single drop of 0.5 % proparacaine hydrochloride (Alcaine; Alcon Laboratories, Inc., Fort Worth, TX, USA) was instilled in the eyes to be operated on to produce a more analgesic effect.

### Surgical procedure

The eyes of the rabbits were cleaned and draped for surgery. The surgical procedure was performed using an operating microscope (Carl Zeiss Meditec AG, Jena, Germany). A lid speculum was placed into the left eye of each animal. A corneal flap (160 μm thick) with a nasal hinge position was made in one eye of each rabbit using the Chiron Automatic Corneal Shaper (Bausch and Lomb Inc, Rochester, NY). A new blade was used in each rabbit. The flap was lifted with a Lindstrom spatula (American Surgical Instruments Corp) and immediately replaced into its original bed. Animals were then divided into three groups (12 eyes each): In group A, fibrin tissue adhesive (Tisseel VH Fibrin Sealant; Baxter Healthcare Corporation, Glendale, CA) was applied to the flap edge using a duploject injector system (Baxter Healthcare Corp., Glendale, CA) after giving a thorough wash to the recipient bed and flap edge to remove the tissue debris, blood clot if any, or any other foreign bodies. The field was then dried with a cellulose acetate sponge. Two sterile microsponges were used to part the wound edges to allow the glue to seep into the base of the flap cut. In group B, the flaps were sutured with 12 interrupted 10/0 nylon sutures (Alcon Laboratories, Fort Worth, TX, USA) placed in 300°. In group C, the flaps were allowed to dry in position for 3 min until it became adherent to the stromal bed without sutures or glue (non-augmented flaps). A bandage soft contact lens was placed over the cornea, and a drop of moxifloxacin (Vigamox, moxifloxacin hydrochloride ophthalmic solution, 0.5 %, Alcon Inc., Dallas, TX) was administered. A tarsorrhaphy using a single 5/0 nylon suture (Alcon Laboratories, Fort Worth, TX, USA) was placed in all eyes and left in place for the first 24 h. All eyes were treated with prednisolone acetate 1 % (Pred Forte 1 %, Allergan, Inc.) and moxifloxacin drops four times a day for 1 week and buprenorphine 0.05 mg/kg, i.m., for 2 days. For the sutured group, the stitches were removed after 3 weeks under general anesthesia.

All rabbits’ eyes were examined using slit-lamp biomicroscopy on postoperative day 1, at week 1, then weekly until the end of the follow-up period.

### Ocular Response Analyzer

The Reichert ORA was used to measure corneal biomechanical parameters for all eyes including unoperated eyes of control group at 6 weeks and 3 months after surgery. The rabbit was adjusted so that the eye is properly facing the noncontact probe after which the device was activated. The noncontact probe of the device emits a rapid air impulse onto the center of the cornea, and sends a signal to the ORA through an optical sensor that measures the deformation of the cornea caused by the air jet. The ORA then displays the corneal hysteresis (CH) and corneal resistance factor (CRF) values on the monitor of the computer attached to the ORA. The ORA software utilized the CH to generate two additional parameters: Goldmann-correlated intraocular pressure (IOPg) and cornea-compensated IOP (IOPcc). The average of four measurements for each eye was taken and those with bad signals or extreme readings were discarded. No cycloplegic eye drops or topical anesthetic were administered before the ORA measurements.

### In vivo confocal microscopy

In vivo confocal microscopy (IVCM) was performed on the corneas of all operated eyes at 6 weeks and 3 months after the surgery using tandem scanning confocal microscopy. Corneas of the unoperated eyes were scanned with the same microscope to differentiate between unoperated corneas under resting state with quiescent keratocytes and operated corneas with activated keratocytes. The eyes were examined with a Tandem scanning confocal microscope (Advanced Scanning Corporation, New Orleans, LA, USA) with a 20× water immersion objective. The confocal microscope had a modified specular objective lens. Light is provided by a remote mercury or xenon lamp. Thus, the microscope and the subject are isolated from the heat and vibration produced by the lamp assembly. This arrangement provides optically sectioned in vivo images with 230× magnification and high resolution. Video sequences were reviewed at least twice and evaluated in a masked fashion. In this study, evaluation of active keratocytes was done in a masked fashion; the differences were documented by 2 independent observers, who agreed completely over the pictures. Under general anesthesia, the rabbit was placed in a supine position in a custom-made resin holder. The microscope objective lens was disinfected with isopropyl alcohol, 70 %, before and after the examination. Methylcellulose was used as an optical coupler between the cornea and the tip of the water-immersion objective. The lens objective was manually advanced until the medium was in contact with the central cornea. A series of confocal images was recorded as the focal plane was advanced manually or automatically from the epithelium posterior to the endothelium. The position of the optical section could be advanced or retracted by an internal lens without changing the position of the front surface of the objective. Images were displayed in real time on a monitor and recorded by a super-VHS recorder through a charge-coupled device video camera onto digital videotape for playback and analysis at a later time.

### Statistical analysis

Statistical analysis was performed using the Student’s t-test and a p value of less than 0.05 was considered statistically significant. Data were expressed as mean ± standard deviation (SD). Calculations were performed using the Statistical Package for the Social Sciences (SPSS) version 18.0 system for personal computers (SPSS Inc., Chicago, IL).

## Results

### Corneal Hysteresis

**Fig. 1:**
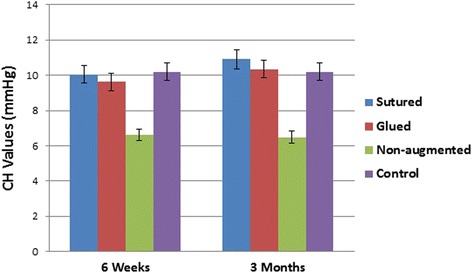
Mean postoperative corneal hysteresis (CH) values for all groups. *P* < 0.0001 for sutured and glued groups vs. non-augmented groups

The mean postoperative CH in glued group (A) was 9.62 ± 0.98 mmHg (range 8.6–11.6 mmHg) and 10.35 ± 0.92 mmHg (range 9–12 mmHg) at 6 weeks and 3 months postoperatively, respectively. In sutured group (B), the mean CH was 10.05 ± 0.85 mmHg (range 8.8–11.3 mmHg) and 10.90 ± 0.95 mmHg (range 9–12.6 mmHg) at 6 weeks and 3 months postoperatively, respectively. In the non-augmented group (C), the mean CH was 6.61 ± 1.18 mmHg (range 5.2–8.1 mmHg) and 6.49 ± 1.20 mmHg (range 5–9.5 mmHg) at 6 weeks and 3 months postoperatively, respectively. In the controls, the mean CH was 10.20 ± 1.17 mmHg (range 8.5–12.5 mmHg) at both 6 weeks and 3 months postoperatively. The mean CH was significantly higher in the sutured and glued groups than in the non-augmented group at all time points (*P* **<** 0.0001). There were no statistically significant differences in mean CH between controls and groups A and B at 6 weeks and 3 months postoperatively (*P* > 0.05; Fig. 1).

### Corneal resistance factor

**Fig. 2:**
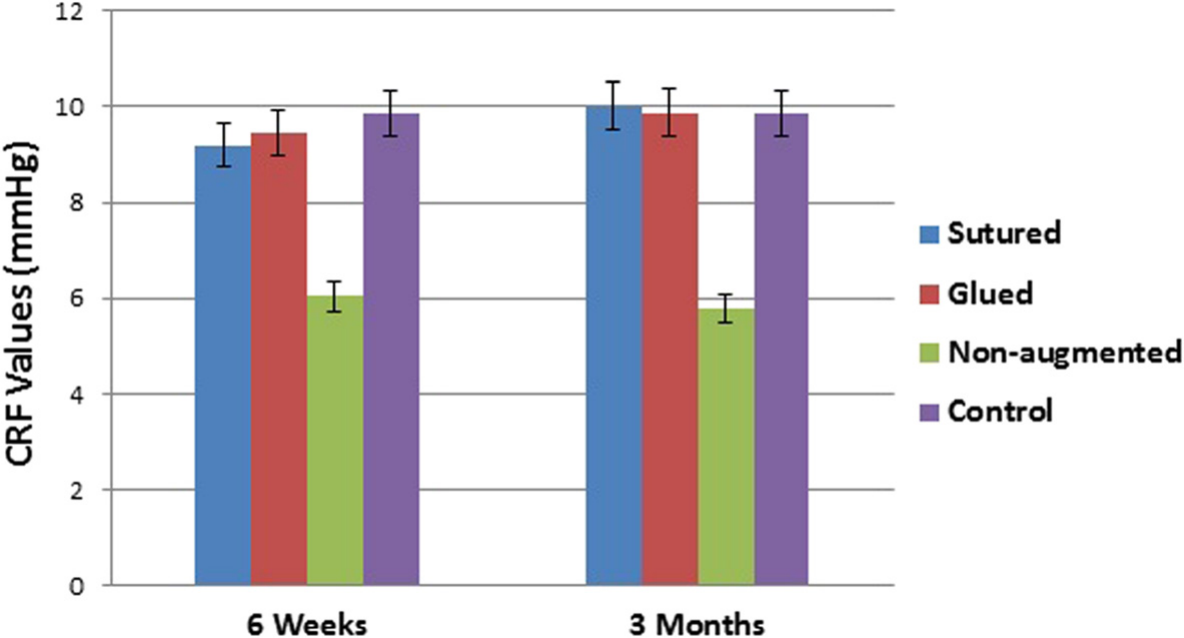
Mean postoperative corneal resistance factor (CRF) values for all groups. *P* < 0.0001 for sutured and glued groups vs. non-augmented groups

The mean postoperative CRF in glued group (A) was 9.45 ± 1.26 mmHg (range 7.5–11.9 mmHg) and 9.87 ± 1.15 mmHg (range 8–12.2 mmHg) at 6 weeks and 3 months postoperatively, respectively. In sutured group (B), the mean CRF was 9.20 ± 1.16 mmHg (range 7.2–11 mmHg) and 10.00 ± 1.14 mmHg (range 8–12 mmHg) at 6 weeks and 3 months postoperatively, respectively. In the non-augmented group (C), the mean CRF was 6.04 ± 1.35 mmHg (range 4.1–8 mmHg) and 5.80 ± 1.00 mmHg (range 4.5–7.5 mmHg) at 6 weeks and 3 months postoperatively, respectively. In the controls, mean CRF was 9.86 ± 0.96 mmHg (range 8.7–12.2 mmHg) at both 6 weeks and 3 months postoperatively. The mean CRF was significantly higher in the sutured and glued groups than in group C at all time points (*P* **<** 0.0001). There were no statistically significant differences in mean CRF between controls and groups A and B at 6 weeks and 3 months postoperatively (*P* > 0.05; Fig. 2).

### Central corneal thickness (CCT), Goldmann-correlated intraocular pressure and Cornea-compensated IOP

At 6 weeks postoperatively, the mean CCT in the sutured, glued, and non-augmented groups was 395.92 ± 7.67 μm, 392.50 ± 12.06 μm, and 401.00 ± 10.37 μm, respectively. In the controls, the mean CCT was 393.14 ± 14.30 μm. At 3 months postoperatively, the mean CCT in the sutured, glued, and non-augmented groups was 389.50 ± 5.50 μm, 390.08 ± 9.33 μm, and 395.66 ± 9.73 μm, respectively. In the controls, the mean CCT was 393.14 ± 14.30 μm.

At 6 weeks postoperatively, the mean IOPg in the sutured, glued and non-augmented groups was 15.30 ± 2.40 mmHg, 14.52 ± 1.11 mmHg, and 15.00 ± 2.63 mmHg, respectively. In the controls, the mean IOPg was 14.73 ± 1.60 mmHg. At 3 months postoperatively, the mean IOPg in the sutured, glued, and non-augmented groups was 15.15 ± 2.00 mmHg, 14.39 ± 1.03 mmHg, and 15.10 ± 2.48 mmHg, respectively. In the controls, the mean IOPg was 14.73 ± 1.60 mmHg.

At 6 weeks postoperatively, the mean IOPcc in the sutured, glued, and non-augmented groups was 15.82 ± 2.44 mmHg, 15.25 ± 1.39 mmHg, and 16.04 ± 2.59 mmHg, respectively. In the controls, the mean IOPcc was 15.35 ± 1.50 mmHg. At 3 months postoperatively, the mean IOPcc in the sutured, glued, and non-augmented groups was 15.94 ± 1.84 mmHg, 15.30 ± 1.18 mmHg, and 15.77 ± 2.64 mmHg, respectively. In the controls, the mean IOPcc was 15.35 ± 1.50 mmHg.

No statistically significant difference was found among all groups in terms of mean CCT, IOPg, and IOPcc at 6 weeks and 3 months postoperatively (*P* ˃0.05). The ORA parameters of all groups are shown in Table 1.

**Table 1:** Summary of corneal biomechanics values at 6 weeks and 3 months for sutured, non-augmented, glued, and control groups

Corneal biomechanics values at 6 weeks and 3 months postoperatively		Sutured	Non-augmented	Glued	Control	*P*-Value*					
						Sutured Vs. Non-aug.	Sutured Vs. Glued	Non-aug. Vs. Glued	Sutured Vs. Control	Glued Vs. Control	Non-aug. Vs. Control
CH (mmHg)	6 weeks	10.05 ± 0.85	6.61 ± 1.18	9.62 ± 0.98	10.20 ± 1.17	<0.0001	0.27	<0.0001	0.5	0.09	<0.0001
	3 months	10.90 ± 0.95	6.49 ± 1.20	10.35 ± 0.92	10.20 ± 1.17	<0.0001	0.11	<0.0001	0.08	0.87	<0.0001
CRF (mmHg)	6 weeks	9.20 ± 1.16	6.04 ± 1.35	9.45 ± 1.26	9.86 ± 0.96	<0.0001	0.61	<0.0001	0.07	0.2	<0.0001
	3 months	10.00 ± 1.14	5.80 ± 1.00	9.87 ± 1.15	9.86 ± 0.96	<0.0001	0.76	<0.0001	0.67	0.98	<0.0001
CCT (μm)	6 weeks	395.92 ± 7.67	401.00 ± 10.37	392.50 ± 12.06	393.14 ± 14.30	0.18	0.42	0.08	0.5	0.9	0.09
	3 months	389.50 ± 5.50	395.66 ± 9.73	390.08 ± 9.33	393.14 ± 14.30	0.07	0.85	0.16	0.4	0.5	0.58
IOPcc (mmHg)	6 weeks	15.82 ± 2.44	16.04 ± 2.59	15.25 ± 1.39	15.35 ± 1.50	0.83	0.48	0.36	0.4	0.8	0.3
	3 months	15.94 ± 1.84	15.77 ± 2.64	15.30 ± 1.18	15.35 ± 1.50	0.85	0.33	0.58	0.3	0.94	0.53
IOPg (mmHg)	6 weeks	15.30 ± 2.40	15.00 ± 2.63	14.52 ± 1.11	14.73 ± 1.60	0.75	0.32	0.58	0.4	0.6	0.7
	3 months	15.15 ± 2.00	15.10 ± 2.48	14.39 ± 1.03	14.73 ± 1.60	0.95	0.26	0.37	0.5	0.52	0.58

*CH* = corneal hysteresis, *CRF* = corneal resistance factor, *CCT* = central corneal thickness, *IOPcc* = cornea-compensated intraocular pressure, *IOPg* = Goldmann-correlated intraocular pressure, *Non-aug.* = non-augmented

*Level of statistical significance *P* < 0.05

### In vivo confocal microscopy

**Fig. 3:**
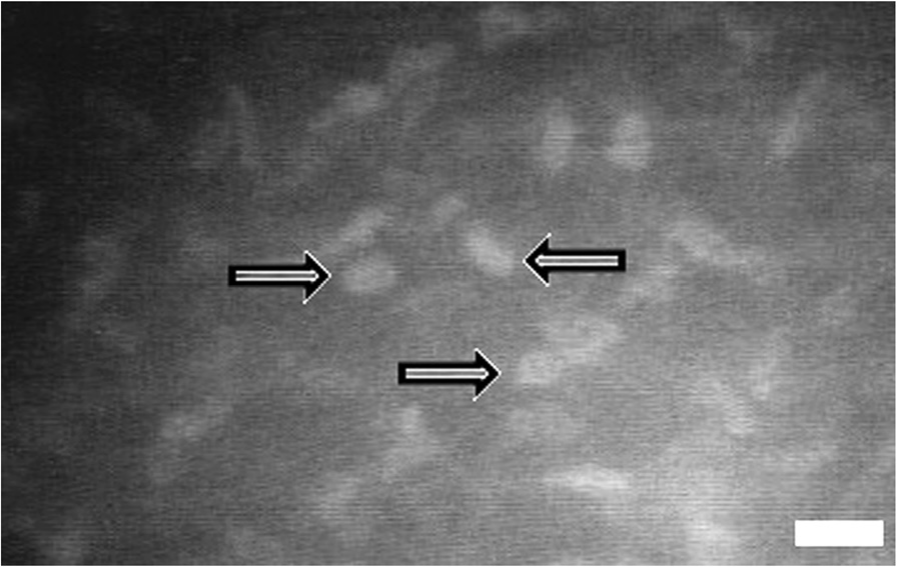
Confocal images of the cornea of unoperated eyes at a depth of approximately 160 μm. Quiescent keratocytes (*arrows*) appeared as bright oval or bean-shaped objects against a dark background. Cellular processes are not evident. Scale bar: 100 μm

**Fig. 4:**
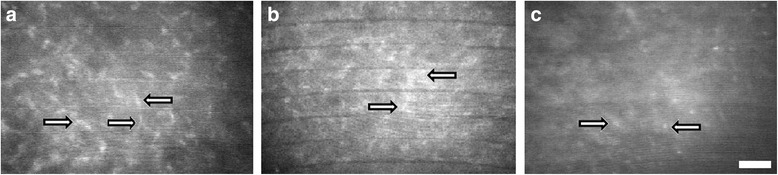
Confocal micrographs of both flap edge and peripheral lamellar interface 6 weeks after surgery. Glued and sutured groups (**a**, **b**): More bright, dense and more reflective activated keratocytes with elongated cell processes (*arrows*) populating the flap edge and the peripheral lamellar interface. Unsutured group (**c**): Less bright and less reflective activated keratocytes with no cell processes (*arrows*). Scale bar: 100 μm

Activated keratocytes have greater corneal light backscattering than quiescent keratocytes during confocal microscopy. As a result, the appearance of activated keratocytes were often large, brightly reflective, and have visible cytoplasmic processes. On the other hand, in the unoperated eyes (control group), quiescent keratocyte nuclei appeared as bright, oval or bean-shaped objects against a dark background. Their cellular processes were not evident in this resting state (Fig. 3). In the glued and sutured groups, many activated keratocytes were found at the peripheral lamellar interface and beneath the flap edge aggregating into a dense cluster mainly in the areas where the previous nylon sutures and adhesive glue were placed. The brightness and reflectivity of activated keratocytes at both the peripheral lamellar interface and beneath the flap edge were more intense in the sutured and glued groups compared with the non-augmented group as revealed by confocal microscopy (Fig. 4). Some of the highly reflective keratocytes appeared considerably larger than others. In all groups, the healing in the central interface was very minimal due to the apposition of two smooth stromal surfaces that resulted in less bright and less reflective activated keratocytes and hence, less haze.

## Discussion

This experiment has shown significantly higher corneal biomechanics in the augmented corneas with sutures or fibrin glues than in the non-augmented flaps, with an impressive durability over time as demonstrated by a significantly higher mean CH and mean CRF in the glued and sutured groups vs. the non-augmented group over the entire follow-up period (*P* < 0.0001). There were no statistically significant differences in mean CH and mean CRF between controls and groups A and B at 6 weeks and 3 months postoperatively.

The strengths of the current study design include in vivo assessments of corneal biomechanical properties and their correlates with the healing process at the lamellar corneal interface and the peripheral flap edge that were augmented with sutures and fibrin glues or were not augmented. Rabbit corneas are approximate models for human corneas concerning biomechanical properties [8].

Increased severing of corneal lamellae is the likely cause of biomechanical instability following LASIK. Collagen lamellae are more densely interwoven in the superficial third of the stroma compared with the deeper two thirds of the stroma. Furthermore, the biomechanical strength of the posterior stroma is less than that of the anterior stroma. Because the load-bearing function of the anterior stroma is disabled after keratotomy, only the weaker, deep stroma is left to maintain corneal integrity [9].

IVCM was used successfully to evaluate in vivo both flap edge and peripheral interface reflectivity and keratocyte status after surgery. Quiescent keratocyte nuclei normally appear by confocal microscopy as bright oval or bean-shaped objects against a dark background. Cellular processes are not evident in the resting state [10]. During confocal microscopy, the activated keratocyte has greater corneal light backscattering than the quiescent keratocyte and their cytoplasmic processes are often visible [11]. In groups A and B, many cells with thick and visible processes, and oval, brightly reflective keratocyte nuclei were seen. These cells were assumed to be activated keratocytes [12]. Many activated keratocytes were found to occupy the peripheral lamellar interface and beneath the flap edge, aggregating into a dense cluster mainly in the areas where the previous nylon sutures and fibrin glue were placed. Activated keratocytes or repair fibrocytes are involved in the production of the repair extracellular matrix that seem to reinforce the wound, thus contributing to the corneal transparency and integrity [13]. The brightness and reflectivity of activated keratocytes were found to be more intense in the sutured and glued flaps compared with the non-augmented ones. As revealed by confocal microscopy, wound healing in sutured and glued groups appeared to come from two sources: the peripheral lamellar interface and beneath the wound edge, while in the non-augmented group, the healing appeared to come only from the central interface. The healing in the central interface in all groups is minimal due to the apposition of two smooth stromal surfaces that resulted in less bright and less activated keratocytes and hence, less haze. The healing area in sutured and glued flaps was therefore more extensive. I hypothesize that this combined healing in groups A and B contributes to integrity of the flap, inducing superior wound strength and hence, better corneal biomechanics in the early postoperative period.

I would venture further to posit that the use of sutures or fibrin glue induces a stronger healing at the corneal flap edge increasing its mechanical strength and may be preventing the late ectasia seen in some LASIK eyes. Several mechanisms for this probably exist. The most likely combination of events is a rearrangement of lamellae, possibly due to altered adhesion, associated with increased activity of degradative enzymes affecting lamellae and ground substance. The use of sutures or fibrin glue may induce a foreign body reaction at the flap edge stimulating an influx of inflammatory cells, a transformation of myofibroblasts and a synthesis of new ground stromal substance.

However, the use of sutures to increase the wound healing response might induce astigmatism, unpredictable refractive results, and prolong surgery time. The current study showed that adhesive fibrin glue can effectively and safely take the place of the sutures to achieve the same results. Cho et al [14] compared wound repair with corneal lamellar grafts in rabbit eyes using human synthetic tissue adhesives and 10-0 nylon and found that human fibrin tissue adhesives were well-tolerated with no apparent corneal toxicity and accelerated wound repair, and can be used as an alternative to sutures in lamellar keratoplasty.

## Conclusions

This study shows that fibrin glue may serve as a safe and effective substitute to sutures in enhancing the corneal flap edge healing response and in increasing its mechanical strength to improve the long term stability of LASIK surgery in borderline thin corneas; however, this must be further corroborated by clinical studies. Compared with non-augmented flaps, glue provides a rapid return of normal corneal biomechanics perhaps because of the combined healing at both the peripheral flap interface and flap margin. A large cohort and longer follow-up may be required to assess the long term efficacy of this procedure.
